# Transparent Ultrathin Metal Electrode with Microcavity Configuration for Highly Efficient TCO-Free Perovskite Solar Cells

**DOI:** 10.3390/ma13102328

**Published:** 2020-05-19

**Authors:** Fengqin He, Hailong You, Xueyi Li, Dazheng Chen, Shangzheng Pang, Weidong Zhu, He Xi, Jincheng Zhang, Chunfu Zhang

**Affiliations:** Wide Bandgap Semiconductor Technology Disciplines State Key Laboratory, School of Microelectronics, Xidian University, Xi’an 710071, China; fq_he@126.com (F.H.); lixueyi9501@163.com (X.L.); dzchen@xidian.edu.cn (D.C.); szpang@stu.xidian.edu.cn (S.P.); wdzhu@xidian.edu.cn (W.Z.); hxi@xidian.edu.cn (H.X.); jchzhang@xidian.edu.cn (J.Z.)

**Keywords:** perovskite solar cells, ultrathin metal electrode, optical microcavity, interference effect, optical coupling layer

## Abstract

Optical microcavity configuration is one optical strategy to enhance light trapping in devices using planar electrodes. In this work, the potential application of optical microcavity configuration with ultrathin metal electrodes in highly efficient perovskite solar cells (PSCs) was investigated. By comparing with the device with conventional indium-tin-oxide (ITO) electrodes, it is shown that by carefully designing the Ag/dielectric planar electrode, a device with an optical microcavity structure can achieve comparable—or even higher—power conversion efficiency than a conventional device. Moreover, there is a relative high tolerance for the Ag film thickness in the optical microcavity structure. When the thickness of the Ag film is increased from 8 to 12 nm, the device still can attain the performance level of a conventional device. This gives a process tolerance to fabricate devices with an optical microcavity structure and reduces process difficulty. This work indicates the great application potential of optical microcavities with ultrathin metal electrodes in PSCs; more research attention should be paid in this field.

## 1. Introduction

Organolead halide perovskites have attracted extensive interest due to their outstanding optoelectronic properties such as tunable compositions, high absorption coefficient, low exciton binding energy, balanced ambipolar carrier transport, long charge carrier diffusion length, solution processing and cost effectiveness [1,2,3,4,5,6,7,8,9]. By improving the film quality, adopting new carrier transporting layers and developing device structures, the power conversion efficiencies (PCEs) of perovskite solar cells (PSCs) in lab-scale devices have improved tremendously from 3.8% to over 22% only within a few years [1,2,3,4,5,6,7,8], approaching those of the long-term established inorganic thin-film solar cells based on Cu(In,Ga)Se_2_ and CdTe [10]. These inherent advantages combining high performance and low-temperature solution processability make PSCs more compatible for scalable roll-to-roll fabrication than their inorganic counterparts [3,11], demonstrating the promising potential for large-scale commercialization [12]. However, to realize the real large-scale applications of PSCs, there are still many problems required to be addressed [13]. For example, PSCs still suffer from the stability issue since the devices are intended to degrade quickly under air condition, which is one big obstacle for the commercialization [1]. With unremitting efforts in the past few years, great progress has been made and the device stability has been greatly improved, which indicates that the reliability of the device is not insurmountable. Besides the device reliability problem, a lower fabrication cost with a higher PCE is the urgently pursued goal in the research and commercialization field of PSCs [1,12]. However, the pursuits of high performance and low cost are two aspects of contradiction. The improvement of the device performance is usually accompanied by the increased cost and we have to balance the device performance and cost. The technology combining the lower cost with the higher device performance, or at least the relative high device performance, is always the pursuit of the research field. Optical microcavity structure could satisfy this requirement [14,15,16].

**Figure 1:**
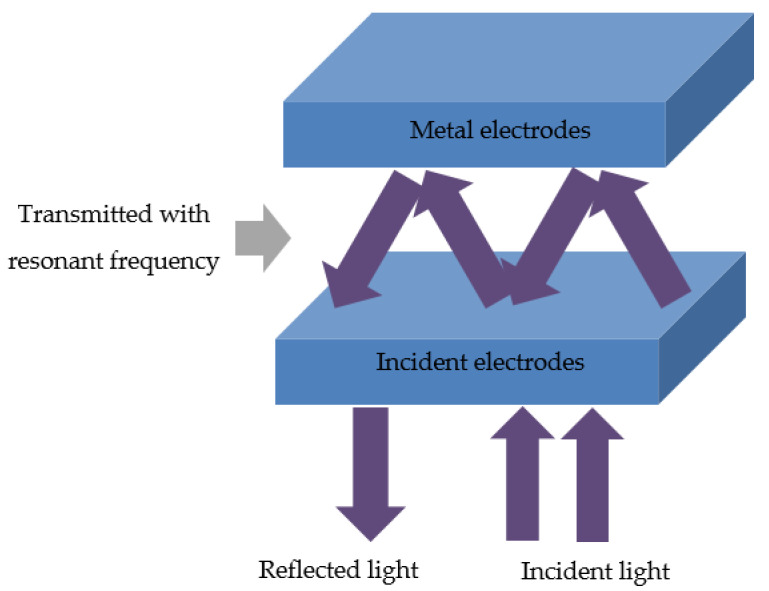
Optical microcavity configuration in solar cells.

Optical microcavity configuration in solar cells is an optical strategy to enhance light trapping in the devices arising from planar electrodes [17]. As is well known, when the thickness of an active layer approaches the wavelength of the visible spectrum in a thin film solar cell, the spatial distribution of the optical electric field within it is determined by the interference of light between the transmitted and reflected waves at each internal interface [18], which in turn affects charge generation within the active layer [19]. To obtain the optical enhancement, solar cells should possess the planar electrodes so that it can reflect the incident light and induce microcavity resonance inside the device as shown in Figure 1. When the resonant frequencies are satisfied, coherent interference occurs, and the incident light will be optically confined and reinforced between two reflective electrodes.

Classically, PSCs possess a transparent electrode and an opaque electrode. Transparent conductive oxides (TCOs) [20], such as indium–tin–oxide (ITO) [21] and fluorine-doped tin oxide (FTO) [22], are the most commonly used transparent electrodes due to their high transparency and relatively low resistivity. However, when ITO or FTO is used in a microcavity configuration, its high transparency limits its potential as a resonant cavity because a large fraction of unabsorbed photons will escape from the device. In this regard, the thin metal film with high reflectivity and transparency could be used instead of TCOs [23].

The adoption of thin metal films has many advantages over TCOs. First of all, its superior reflective properties compared to TCOs (such as ITO) could ensure the formation of a stronger microcavity and confine more light inside the device [24]. Furthermore, the conductivity of thin metal films is very higher than TCOs, which could reduce the series resistance and is very important for the large area devices [25,26]. In addition, the adoption of thin metal films possesses the potential to lower the fabrication cost. For example, the widely used ITO have the component of indium which is a scarce and toxic material, and thus the highest share in material cost and energy input of ITO is inevitable in the fabrication process, while the metal deposition process is very mature and widely used in the industry [21,27]. In addition, from a structural point of view, the TCO itself is a brittle ceramic structure that limits its flexibility. When the TCO electrode is bent to a certain extent, it will inevitably introduce defects, affecting the device performance [20]. Metal has ductility and flexibility, which makes it very suitable for the use in the flexible devices. However, the thin metal films also have the obvious disadvantage. They exhibit strong absorption and reflection in the visible and near infrared spectroscopy for its inherent characters so that some incident light cannot pass the thin metal film electrode and reach the active layer [28]. That is to say, although the superior reflectivity of thin metal film could ensure the formation of a stronger microcavity, it can also induce the light loss. Moreover, the dual characteristics must be carefully balanced. To enhance the transmittance and at the same time obtain an effective mirror-like thin metal film [29,30], the uniformity, thickness, reflective and transparent properties of the metal film must be controlled carefully [31]. The combination of metal/dielectric could be an effective structure to adjust the optical properties of thin metal film [32].

By using metal/dielectric structure, the optical microcavity configuration with the ultrathin metal electrode has been used in organic solar cells and achieves promising device performance [33]. However, the reports about optical microcavity in PSCs are relative few. One possible reason may be that in the past few years, PSCs developed very fast and most work focuses on the PSCs themselves such as the perovskite material and the transporting layers, and few people pay attention to the optical management. With the further development, more research attention should move to the optical management. Another reason for few reports of optical microcavity in PSCs may be that people are not exactly sure if the optical microcavity is still effective for PSCs. The active layer in organic layer has a thickness of several tens of nanometers or around one hundred nanometers. However, the thickness of active layer in PSCs is usually several hundred nanometers. In addition, the perovskites have also a wider absorption range than the organic materials. Both of that are sensitive to the optical microcavity resonance inside the device. Whether optical microcavity can improve the device performance or not should be further clarified and this is the goal of this work. We will show that optical microcavity configuration with the ultrathin metal electrode is still an effective strategy to enhance the device performance in PSCs and more research attention should be paid in this field.

## 2. Theory and Method

When the active layer is sandwiched by two reflective electrodes in solar cells, the optical microcavity could be formed as shown in Figure 1. In order to achieve a strong resonance in a microcavity structure, the thickness of active layer should be comparable or smaller than the wavelength of visible light. When the film thickness increases, the coherent interference will become to be weak. The interference could be constructive or destructive. In order to achieve constructive interference within the device, the two reflective electrodes should be at the nodes of the optical electric field. For a given wavelength (λ), the optically resonant condition can be expressed by Equation (1):

(1) $$\underset{i}{\sum }n_{i}d_{i}+\left(\varphi_{1}+\varphi_{2}\right)\frac{\lambda }{4\pi }=\frac{m\lambda }{2}$$

where n_i_ is the real part of the refractive index in each layer between the two reflective electrodes, d_i_ is the corresponding thickness of each layer, φ_1_ and φ_2_ are the respective phase changes due to the reflection at each interface, and m is a positive integer.

The left part of Equation (1) consists of two parts. One part is the optical length of the chamber and the other part is the phase change of the field induced by reflection at each electrode. From Equation (1), considering the phase change, constructive interference can be obtained when the optical path length equals to a multiple integer of the half wavelength of the incident light. Once constructive interference is built, the optical microcavity can trap the incident light. However, Equation (1) is not tenable for any wavelength of incident light due to the fixed geometric parameters of the optical microcavity. Only a narrow spectral range of incident light with resonant frequencies can be trapped in the microcavity. Therefore, the optical field distribution inside the devices need to be carefully controlled so that the light absorption in the devices could be maximized.

**Figure 2:**
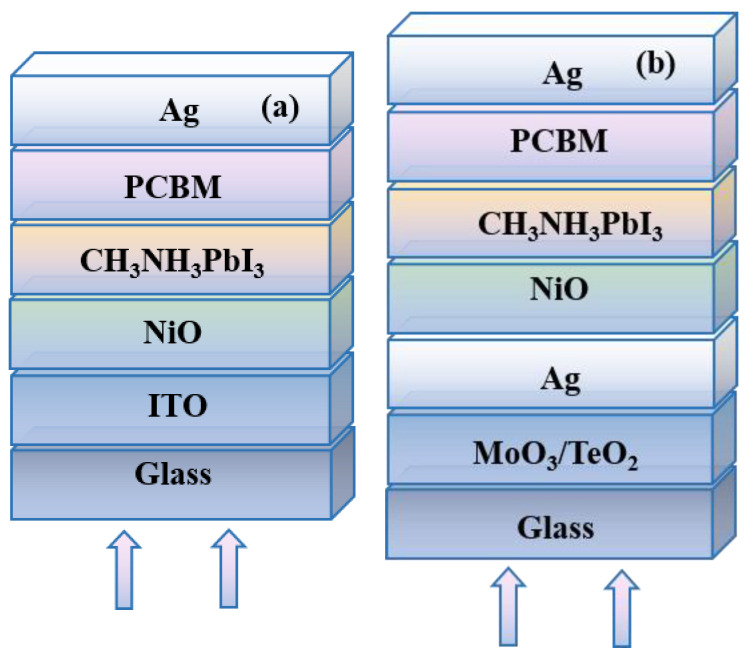
The structural models for (**a**) conventional structure (D1) and (**b**) optical microcavity structure (D2).

To clarify the function of optical microcavity in PSCs, the distribution of the optical electric field and the electrical properties of devices are obtained by the transfer matrix method and drift–diffusion equations. The calculation method is detailed introduced in the supplementary information. Several assumptions are used in the calculations, for example, every layer is homogenous and uniform, interfaces are parallel and flat compared to the wavelength of the light and the light in the device can be described by plane waves. In some materials, the anisotropy of the refractive index could affect the device performance greatly [34]. However, this is not an important issue in perovskite materials. For simplification, the perovskite material is regarded as an isotropic material, and no anisotropy of the refractive index is considered in this study. The normally incident AM 1.5 G solar spectrum is chosen as light source; optical constants (refractive index of the materials) and the layer thicknesses are used as initial parameters. All the refractive index of the materials is shown in Figure S1 in the supplementary information. The inverted planar structure is used in this work. As shown in Figure 2, the typical CH_3_NH_3_PbI_3_ is chosen as the active layer, NiO acts as the hole transport layer, and the phenyl-C_61_-butyric acid methyl ester (PCBM) play the role of electron transport layer (ETL). The device with the traditional ITO electrode acts as the reference device (D1 in Figure 2a). The device with optical microcavity based on the semitransparent silver thin film (D2 in Figure 2b) is investigated here.

In the device of D2, the opaque Ag and ultra-thin Ag film form the optical microcavity, and the high dielectric MoO_3_ or TeO_2_ is selected as the optical coupling layer to adjust the transmittance of ultrathin Ag electrode. The devices are illuminated from the bottom direction. The calculation details and the used parameters could be found in the supplemental information.

## 3. Results and Discussion

**Figure 3:**
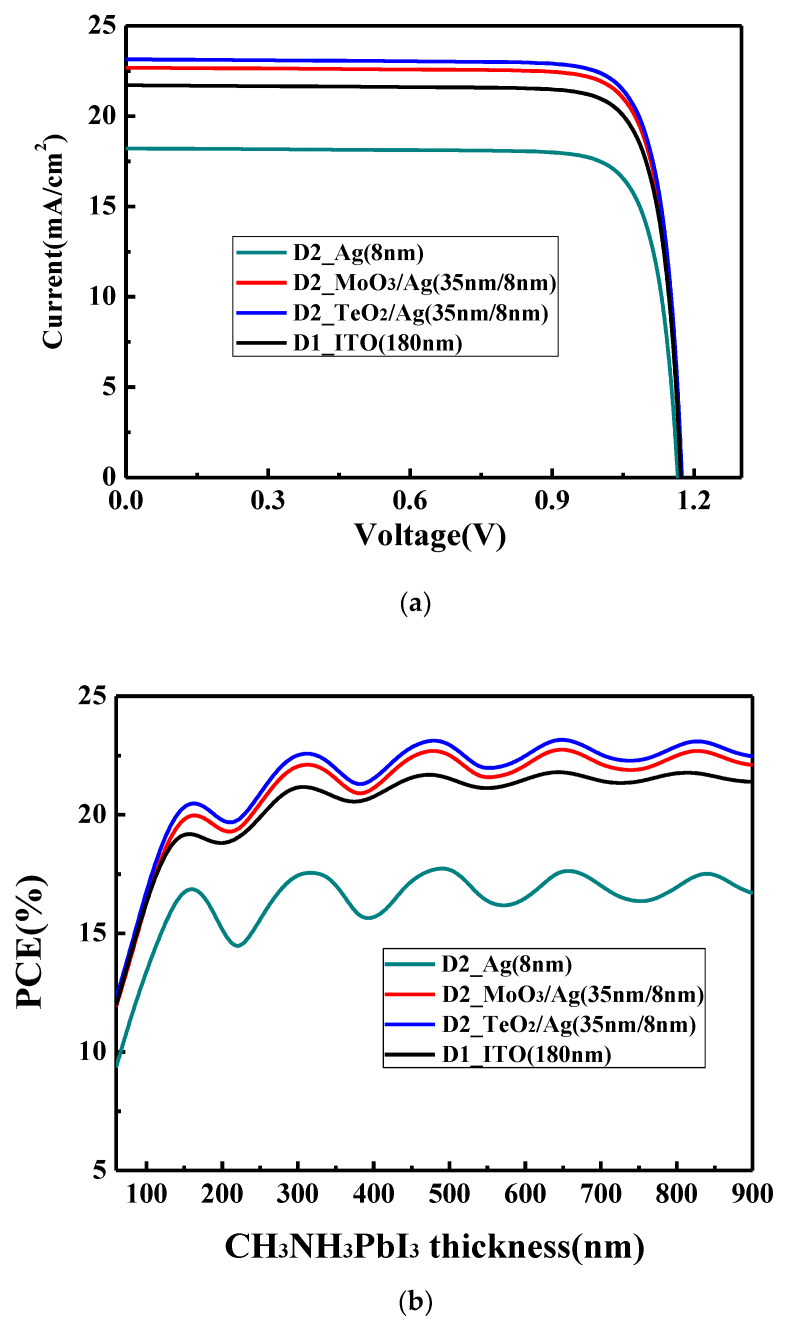
(**a**) I–V curves for device D1 and device D2 and (**b**) variation of power conversion efficiency (PCE) for device D1 and device D2 with various CH_3_NH_3_PbI_3_ thicknesses.

Using the method described in our previous publications [35,36,37,38] and also stated in the supplemental information, we first calculate the current–voltage (I–V) characteristics of PSCs based on the conventional ITO electrode to verify the validity of our calculation method in this work. As shown in Figure 3a, the device with the structure of glass/ITO (180 nm)/NiO (30 nm)/CH_3_NH_3_PbI_3_ (320 nm)/PCBM (45 nm)/Ag (100 nm) shows a PCE of 21.1% with short circuit current density (J_SC_) of 21.7 mA/cm^2^, open circuit voltage (V_OC_) of 1.17 V and fill fact (FF) of 83.2%. Many reports [1,2,5,6,7] has shown that the J_SC_ can get the value between 20 and 24 mA/cm^2^, V_OC_ can reach a value larger than 1.1 V, FF can obtain a value between 80% and 84% and PCE could be higher than 20% (the reported highest PCE is around 25%) in the experiments. Our calculated values fall in this range and the obtained parameters are reasonable. This confirms that the method to calculate the device characteristics is effective and feasible, which will be used in the following discussion.

The variation of PCE with the perovskite layer thickness for the device D1 on the ITO electrode is shown in Figure 3b. In general, PCE increases with the active layer thickness, implying more thickness will give better efficiency. This is different from the organic solar cells where a limited thickness is usually observed [39]. In organic solar cells, the carrier mobility is very low and when the thickness increases, some carriers cannot be collected efficiently by the electrode due to the recombination, which limit the optimal active layer thickness. However, this is not an issue for PSCs which have a much higher charge mobility and relative longer carrier lifetime. Moreover, thus the recombination is not the limited factor in the thickness range of investigation here and this explains why the general trend in Figure 3b is that PCE increases with the active layer thickness. It can also be observed that with the thickness increase of the perovskite layer, PCE shows the oscillation, which is the obvious feature of the optical interference. Figure S2 shows that when the thickness of the perovskite layer increases, the changes of V_OC_ and FF is relatively small, while there is an obvious change for J_SC_. It is well known that J_SC_ is tightly related to the light absorbability of the perovskite layer. If the optical interference can be neglected, a thicker perovskite layer can absorb more light and there will be no oscillation for J_SC_. However, the obvious J_SC_ oscillation in Figure S2 shows that the optical interference must be considered. As is well known, the visible light region is one region with the most energy in the solar spectrum and the perovskite layer mainly absorbs the light in this region (below 800 nm). The thickness of the perovskite layer used in the calculation is from 50 to 900 nm, which is near to the wavelength of the visible light and under this condition it will lead to the optical interference as stated in the theory part. In the microcavity structure, we require a mirror-like electrode. However, the reflectivity of ITO is weak, and it is not a good choice as an electrode in the microcavity structure. Thus, we need to choose another mirror-like material such as the metal and carefully design the microcavity structure. Before moving to the microcavity structure design, we noted that in Figure 3b, the second oscillation peak appears when the thickness of the perovskite layer is around 320 nm and a thicker perovskite layer almost can hardly improve the device performance further. The thickness of the perovskite layer prepared by the one-step method is often in the range of 300 to 350 nm [6,7,9] and it is just around the second oscillation peak. Thus, the 320 nm thickness of the perovskite layer will be used in the following.

**Figure 4:**
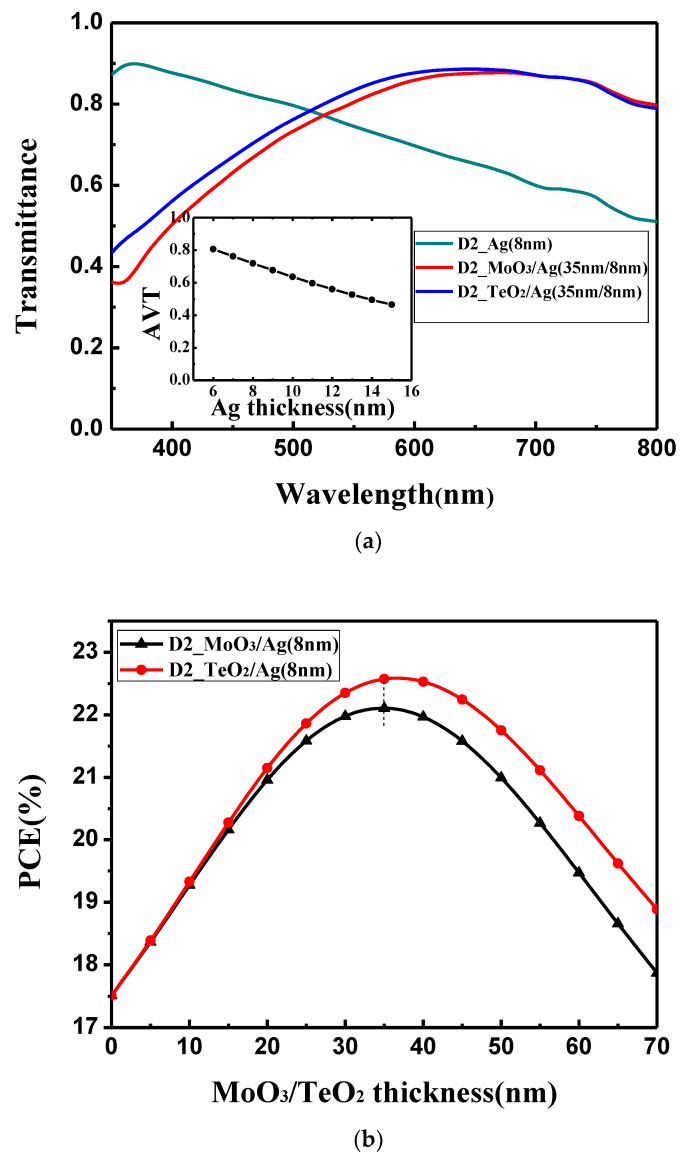
(**a**) Optical transmittance for Ag (8 nm), MoO_3_ (35 nm)/Ag (8 nm) and TeO_2_ (35 nm)/Ag (8 nm) with various light wavelength. Inset: the average transmittance for different Ag thicknesses; (**b**) variation of the calculated PCE with the different thicknesses of MoO_3_ and TeO_2_.

To enhance the interference resonance in the microcavity structure, a mirror-like metal can be used such as Ag, Au, Al, etc. However, because light will enter the device across this electrode, the electrode should also possess a relative high transmission in the visible reason so that more light could reach the active layer. Thus, there is a balance between the reflectivity and transmittance for the electrode. By comprehensively considering the conductivity, absorption in the visible region and cost-effectiveness, Ag is chosen as the electrode to take place of ITO. The percolation threshold of thermally evaporated Ag film can be decreased from 11 to 9 nm by the modifying surface energy in our laboratory [40] and the thinner 8 nm was experimentally reported [41]. Therefore, the ultrathin 8 nm Ag film is chosen as the electrode and the calculated I–V curve is also shown in Figure 3a. By using the bare 8 nm Ag, the device shows a PCE of 17.6% with J_SC_ of 18.2 mA/cm^2^, V_OC_ of 1.16 V and FF of 82.9%. It is obviously lower than the device with the ITO electrode where 21.1% PCE is obtained. As shown in Figure 3b, when the 8 nm Ag film is used, PCE shows an obvious oscillation, which is similar with the device with the ITO electrode. It should be noted the oscillation period and the points where the oscillation peaks achieved are the same for the devices with Ag electrode and ITO electrode. This is because the geometry size of the microcavity is the same for both the devices. However, what needs to be pointed out is that the oscillation amplitude for the device with the Ag electrode is obviously larger than that with the ITO electrode. This means that the mirror-like Ag electrode could effectively trap the light in the microcavity, and the resonance phenomenon is more obvious. Thus, an enhanced optical interference resonance could be observed in this device. Although there is a more obvious resonance phenomenon, the overall device performance is still low. This is because more light cannot reach the active layer. As shown in Figure 4a, 8 nm Ag film shows an obviously inferior light transmittance. The average transmittance value between 350 and 800 nm is below 70%, which is lower than the 180 nm ITO whose average transmittance value between 350 and 800 nm is around 84%. We must balance the reflectivity and transmittance of the Ag electrode. Thus, we try to change the optical properties of the Ag thin film by using the optical coupling layer.

We choose MoO_3_ and TeO_2_ as the optical coupling layer to enhance the optical transmittance of the Ag film electrode due to their large dielectric constant (ε). Figure S3 and Figure 4b show the variation of the calculated V_OC_, FF and PCE with the thickness of the dielectrics. After the optical coupling layer is added, V_OC_ and FF keep almost constant as in Figure S3. However, with increasing thickness of the MoO_3_ and TeO_2_, the PCE increases first and then decreases, and both get the optimal value at 35 nm. The reason is that if the optical coupling layers are too thin, they cannot improve the optical transmittance of the Ag film electrode in the long wavelength; if coupling layers are too thick, they will affect the optical transmittance of the Ag film electrode in the short wavelength. This can also be seen from Figure 4a that the optical transmittance of the bare Ag electrode is higher than that with the optical coupling layer in the short wavelength, and the transmittance of the bare Ag electrode is lower than that with the optical coupling layer in the long wavelength. Comparing the functions of MoO_3_ and TeO_2_, it shows that the optical transmittance of TeO_2_ /Ag (35 nm/8 nm) is slight higher than that of MoO_3_/Ag (35 nm/8 nm) in the wavelength range of 350 to 700 nm because a higher dielectric layer (εTeO_2_ = 25) can suppress a large optical dissipation related to the emission of non-radiative surface plasmons at the interface of dielectric layer and Ag film (ε = 9.7) [42,43,44]. When the optical coupling layer is at the optimal thickness (35 nm), the overall optical properties of the dielectric/Ag electrode is the best ant the device obtains the highest PCE. The optimized I–V curves are shown in Figure 3a. For the device with the MoO_3_/Ag electrode, a PCE of 22.2% with J_SC_ of 22.8 mA/cm^2^, V_OC_ of 1.17 V and FF of 83.3% is obtained. For the device with the TeO_2_/Ag electrode, a higher PCE of 22.6% with J_SC_ of 23.2 mA/cm^2^, V_OC_ of 1.17 V and FF of 83.2% is obtained. Using the dielectric/Ag electrode, the obvious oscillation with the same period and peak wavelength as the ITO electrode is also observed. In addition to the dielectric, the oscillation amplitude for the device is now between the amplitude of the ITO electrode and that of the bare Ag electrode. This shows that the balance between the reflectivity and transmittance for the electrode was obtained. By designing the microcavity, we surprisingly find although less light can pass the dielectric/Ag electrode and reach the active layer compared with the device with the ITO electrode, the device still achieves a higher performance because more light would be trapped in the microcavity.

**Figure 5:**
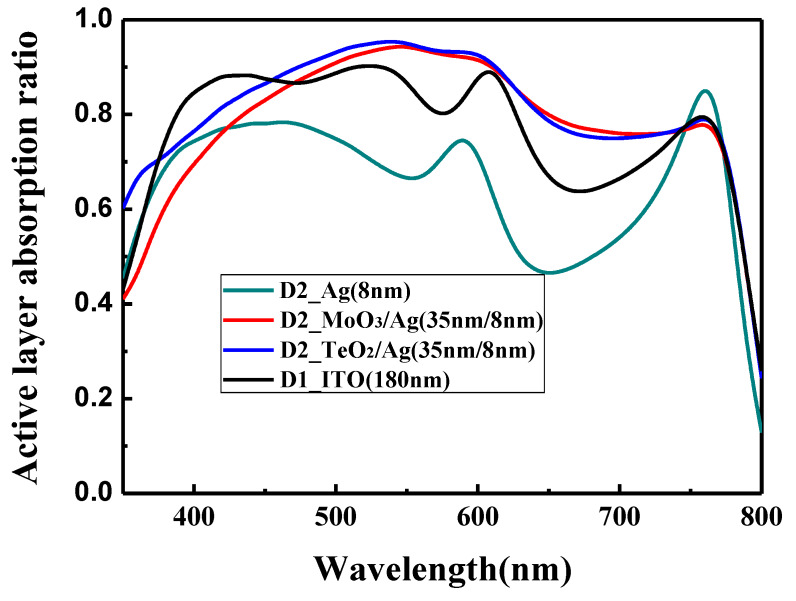
Active layer absorption ratio for device D1 and device D2 with various wavelengths.

The calculated optical absorption ratio at different wavelengths of the perovskite active layer in the conventional structure and the optical microcavity structure is shown in Figure 5. All the devices have the same thickness of the perovskite active layer (320 nm). However, they show different light absorption ability. For the conventional device with the ITO electrode, the absorption ratio is good in the wavelength of 380 to 620 nm. Beyond 620 nm, there is a decline for the light absorption, which may be induced by the low light trap ability of ITO for the long wavelength. For the bare Ag incident electrode, the absorption ratio is the lowest, and the absorption mainly concentrates in the range of 400–600 nm, which is due to the low light transmittance of the Ag film. For the MoO_3_/Ag (35 nm/8 nm) incident electrode and the TeO_2_/Ag (35 nm/8 nm) incident electrode, the absorption is high in the wavelength range of 400–750 nm, which is slightly higher than the ITO electrode. This confirms the validity of the optical microcavity structure to improve the effective absorption ability of PSCs.

**Figure 6:**
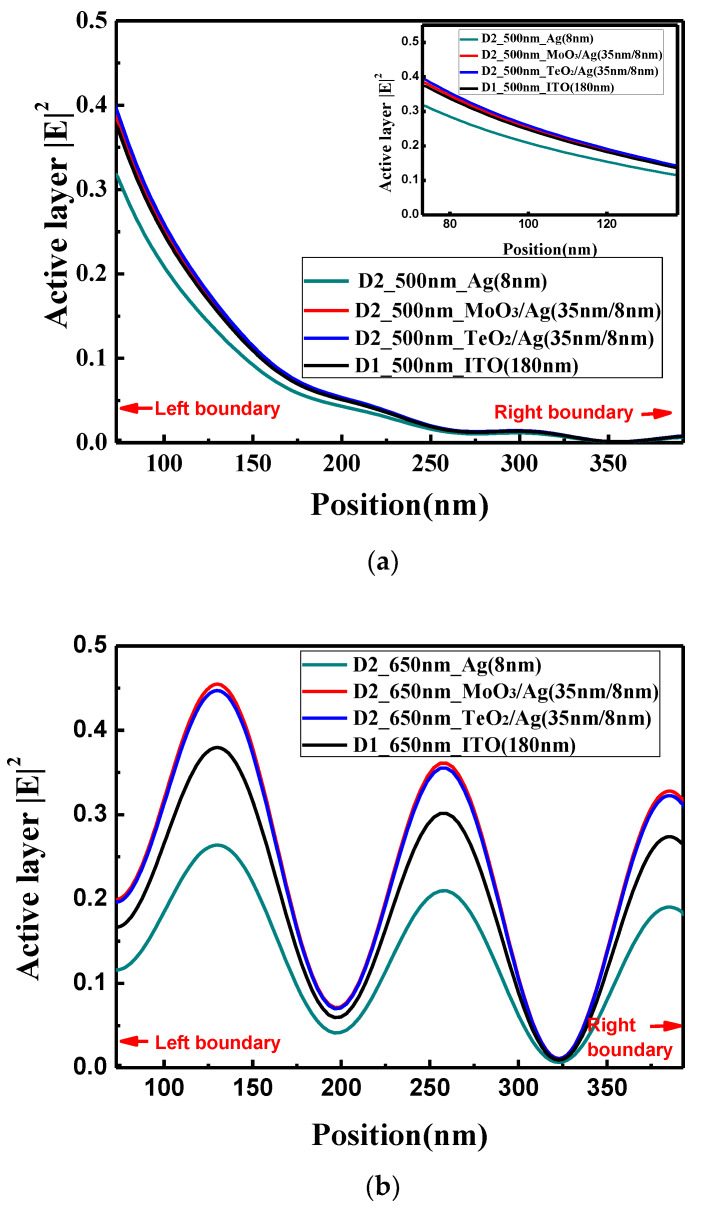
(**a**) Optical electric field distribution at 500-nm wavelength in the perovskite active layer; (**b**) optical electric field distribution at 650-nm wavelength in the perovskite active layer. Red arrows in the figure indicate the left and right boundaries of the perovskite layer.

The optical electric field distributions in the perovskite active layer at wavelength of 500 and 650 nm are shown in Figure 6a,b, respectively. When the light wavelength is 500 nm, there is no obvious interference peaks in the active layer. The optical electric field of the device with the bare Ag electrode is the lowest, and the optical electric field of TeO_2_/Ag, MoO_3_/Ag and ITO decrease in sequence, which is consistent with the absorption rate in the active layer. However, the absorption difference among the devices with TeO_2_/Ag, MoO_3_/Ag and ITO electrodes is very small. When the light wavelength is 650 nm, the obvious interference peaks are observed. The optical electric field for the device with the bare Ag electrode is also the lowest. Compared with the conventional device with the ITO electrode, the optical electric field in the microcavity structure with the TeO_2_/Ag, MoO_3_/Ag electrode is obviously larger, which shows the advantages of the optical microcavity structure, which partially verifies that the careful design of the optical microcavity could improve the performance of PSCs.

**Figure 7:**
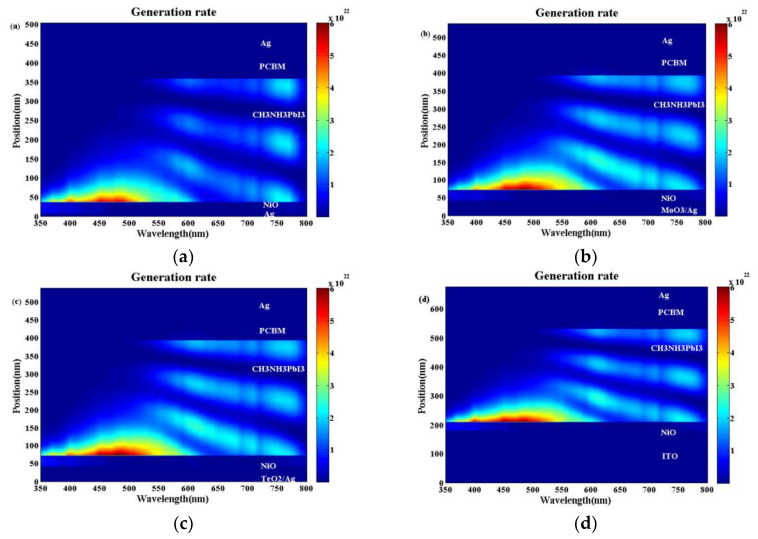
Exciton generation profiles for optical microcavity perovskite solar cells (PSCs) with (**a**) bare Ag (8 nm) electrode, (**b**) MoO_3_/Ag (35 nm/8 nm) electrode exciton generation rate, (**c**) TeO_2_/Ag (35 nm/8 nm) electrode and (**d**) conventional PSC.

The exciton generation profiles for the conventional device and the optical microcavity device are shown in Figure 7. Moreover, the corresponding optical electrical field distributions are also shown in Figure S4. It can be seen from Figure 7a–d that for all the devices, when the light wavelength is shorter than 550 nm, the intense constructive optical interference cannot be built. The optical electric field and the exciton generation rate decrease monotonically when the light enters the perovskite active layer. This is why there is no interference peaks when the light wavelength is 500 nm as shown in Figure 6a. When the light wavelength is longer than 550 nm, obvious optical interference could be built from Figure 7a–d. Moreover, the longer the light wavelength, the stronger the optical interference is. Compared with the bare Ag electrode in Figure 7a and Figure S4a, we can see there is a much stronger optical electric field at the interference peaks as in Figure 7b,c and Figure S4b,c. When the TeO_2_/Ag and MoO_3_/Ag electrodes are used, which approaches or even exceeds the case with the ITO electrode as shown in Figure S4b,c. Correspondingly, there is a larger exciton generation rate for the optical microcavity structure with the TeO_2_/Ag (Figure 7c) or MoO_3_/Ag (Figure 7b) electrodes than the bare Ag electrode (Figure 7a) and even better than that with the ITO electrode as in (Figure 7d). The much larger exciton generation rate will contribute a higher J_SC_ and PCE, which is why the devices with the TeO_2_/Ag or MoO_3_/Ag electrodes have the better device performance than the conventional device with the ITO electrode as shown in Figure 3.

In the above discussion, we considered the amplitude of optical electric field and the total exciton generation number in Figure 6 and Figure 7. In fact, in some solar cells such as the organic solar cells, the exciton generation position is also important. The reference [39] has discussed this issue and they suggested if electrons and holes have the same mobility, it is better to generate exciton in the center of the active layer. This is because there is a very low electron and hole mobility in organic solar cells and some charges will recombine before they can reach the electrodes. Thus, when the generate exciton is at the center of the active layer, the balanced hole and electron collection could be achieved. However, the electron and hole mobility in PSCs is much higher than in organic materials. In addition, the perovskite layer thickness here is relatively low and usually several hundred nanometers. Almost all the charges can reach the electrodes before they recombine. Thus, the exciton generation position is not the limited factor and the total exciton generation number is more important.

We have shown that the optical microcavity structure could be used in the PSCs to achieve a higher efficiency than that with the conventional ITO electrode by adopting the TeO_2_/Ag or MoO_3_/Ag electrodes. In above discussion, we use an Ag thickness around 8 nm. However, it is not easy to achieve such an ultrathin Ag film. Compared with this, a thickness around 10 nm or thicker Ag film is much easier to be obtained. Thus, we further investigated the application robust of the optical microcavity in PSCs by using a thicker Ag film. The results are shown in Figure S5 for the 10-nm-thick Ag film and Figure S6 for the 12-nm-thick Ag film. All the devices with the optical microcavity show the same trend as that using 8-nm Ag. Figure S7 shows that even when the thickness of the Ag film was as great as 12 nm, the device with the optical microcavity structure still could achieve a PCE over 20% and approach the performance with the ITO electrode. This shows the applicability of the optical microcavity in PSCs.

## 4. Conclusions

In this work, we investigate the potential use of the optical microcavity with the ultrathin metal electrode in PSCs. By comparing with the conventional ITO electrode, it is shown that by carefully designing the Ag/dielectric electrode, the device with the optical microcavity structure can achieve a higher PCE than the conventional device. The results show that the bare Ag metal electrode has poor light transmittance, the corresponding device has a poor absorption rate and low PCE, and then the bare Ag metal is not suitable in the optical microcavity. The addition of MoO_3_ and TeO_2_ optical coupling layer significantly improves the light transmittance of the incident electrode, improves the absorption in the perovskite active layer, increases the optical electric field and exciton generation rate, and results in a very high PCE. When the thickness of CH_3_NH_3_PbI_3_ was 320 nm, the PCE of the device with the TeO_2_/Ag (35 nm/8 nm) electrode was the highest, reaching 22.60% and 1.45%, higher than the conventional device. Moreover, the optical microcavity structure was robust when there was a much thicker Ag film. When the thickness of the Ag film was increased to 12 nm, the device could still achieve the level of performance as the conventional device. This gives a process tolerance to fabricate the device with the optical microcavity structure and reduces process difficulty. In summary, this work indicates the great application potential of the optical microcavity with the ultrathin metal electrode in PSCs and more research attention should be paid in this field.

## Supplementary Materials

The following are available online at https://www.mdpi.com/1996-1944/13/10/2328/s1, Calculation method, Figure S1: Refractive index of the materials used in the calculation; Figure S2: Variation of Jsc, Voc, FF for device D1 and device D2 with various CH_3_NH_3_PbI_3_ thicknesses; Figure S3: Variation of the calculated V_OC_ and FF with the different thicknesses of MoO_3_ and TeO_2_; Figure S4: The optical electric field distribution for optical microcavity PSCs with (a) bare Ag (8 nm) electrode, (b) MoO_3_/Ag (35 nm/8 nm) electrode, (c) TeO_2_/Ag (35 nm/8 nm) electrode and (d) the conventional PSC with the ITO electrode; Figure S5: Variation of Jsc, Voc, FF and PCE for device D1 and device D2 with various CH_3_NH_3_PbI_3_ thicknesses when the thickness of Ag is 10 nm; Figure S6: Variation of Jsc, Voc, FF and PCE for device D1 and device D2 with various CH_3_NH_3_PbI_3_ thicknesses when the thickness of Ag is 12 nm; Figure S7: I–V characteristic curves for the optical microcavity devices with the 10 nm Ag (a) and the 12 nm Ag (b); Table S1: The parameters used in the calculation.
