# Supplementary material for: Transparent Ultrathin Metal Electrode with Microcavity Configuration for Highly Efficient TCO-Free Perovskite Solar Cells
Source: Materials (Basel). 2020 May 19;13(10):2328. doi: 10.3390/ma13102328 (PMC7287901; doi:10.3390/ma13102328)
Supplement: Supplementary file 1 [file materials-13-02328-s001.pdf]

*materials*

*Supplemental information*

# Transparent Ultrathin Metal Electrode with Microcavity Configuration for Highly Efficient TCO-Free Perovskite Solar Cells

**Fengqin He, Hailong You *, Xueyi Li, Dazheng Chen, Shangzheng Pang, Weidong Zhu, He Xi, Jincheng Zhang and Chunfu Zhang ***

Wide Bandgap Semiconductor Technology Disciplines State Key Laboratory, School of Microelectronics, Xidian University, Xi'an 710071, China; fq_he@126.com (F.H.); lixueyi9501@163.com (X.L.); dzchen@xidian.edu.cn (D.C.); szpang@stu.xidian.edu.cn (S.P.); wdzhu@xidian.edu.cn (W.Z.); hxi@xidian.edu.cn (H.X.); jchzhang@xidian.edu.cn (J.Z.)

* Correspondence:hlyou@mail.xidian.edu.cn (H.Y.); cfzhang@xidian.edu.cn (C.Z.)

Received: 13 April 2020; Accepted: 14 May 2020; Published: date

**Calculation Method:**

In the optical calculation, the TMF method is used as in our previous reports (Chinese Phys. B, 18:2096, 2009; Chinese Phys. B, 18: 349, 2009; Jpn. J. Appl. Phys., 51: 122301, 2012; IEEE Transactions on Electron Devices, 60(1): 451–457, 2013). Only light at normal incidence to the substrate is considered for photovoltaic devices. It is assumed that every layer is flat and homogenous, and one absorbed photon produces one electron-hole pairs (one electron and one hole).

To calculate the number of absorbed photons (or excitons) in the active layer, the energy flow dissipation per second at single wavelength in the active layer, Q is given as

$$Q = \frac{1}{2} c\varepsilon_0 \alpha n |E|^2 \quad (1)$$

where c is the speed of light in vacuum, ε0 the permittivity of vacuum, $\alpha$ the absorption coefficient, n the real index of refraction and $|E|^2$ the total optelectric field intensity in the multiplayer stack at single wavelength. Then the number of photons absorbed in the active layer can be expressed as

$$N = \int_{\lambda=300nm}^{\lambda=800nm} Q(\lambda) \frac{\lambda}{hc} d\lambda \quad (2)$$

where N represents the number of absorbed photons, hc/$\lambda$ the photon energy at a specified wavelength $\lambda$, h the Planck constant and c the speed of light in vacuum. From the obtained N, the carriers generation rate G could be obtained.

For the electrical properties, the calculation is mainly based on three basic equations which are Poisson's equation, carrier continuity equation and drift-diffusion equation, respectively. For this part, the commercial software (Silvaco) is used.

Poisson's equation:

$$\frac{\partial^2 \varphi}{\partial x^2} = \frac{q}{\varepsilon}(n - p) \quad (3)$$

Carrier continuity equation:

$$\frac{\partial n}{\partial t} = \frac{1}{q}\frac{\partial J_n}{\partial x} + G - R \quad \frac{\partial p}{\partial t} = -\frac{1}{q}\frac{\partial J_p}{\partial x} + G - R \quad (4)$$

Drift-diffusion equation:

$$J_n = qD_n \frac{\partial n}{\partial x} - q\mu_n n \frac{\partial \varphi}{\partial x} \quad J_p = -qD_p \frac{\partial p}{\partial x} - q\mu_p p \frac{\partial \varphi}{\partial x} \quad (5)$$

where $\varphi$ is electric potential, $\varphi$ is dielectric constant, q is electron charge, n is electron concentration, p is hole concentration, Jn is electron current density, $J_p$ is hole current density, G is carriers generation rate, R is carriers recombination rate, $D_n$ is electron diffusion coefficient, $D_p$ is hole diffusion coefficient, $\mu_n$ is electron mobility, $\mu_p$ is hole mobility. The used parameters are as the following (Table S1)

**Table S1.** Parameters used in the calculation. PCBM is the phenyl-$C_{61}$-butyric acid methyl ester.

| Parameters | PCBM | Perovskite |
|---|---|---|
| Thickness (nm) | 40 | 320 |
| Eg (eV) | 1.8 | 1.5 |
| Permittivity | 4.0 | 30 |
| Affinity (eV) | 4.1 | 3.9 |
| Nc ($cm^{-3}$) | $2.5 \times 10^{19}$ | $1 \times 10^{20}$ |
| Nv ($cm^{-3}$) | $2.5 \times 10^{19}$ | $1 \times 10^{20}$ |
| Mun ($cm^2 V^{-1} s^{-1}$) | 0.01 | 50 |
| Mup ($cm^2 V^{-1} s^{-1}$) | 0.01 | 50 |

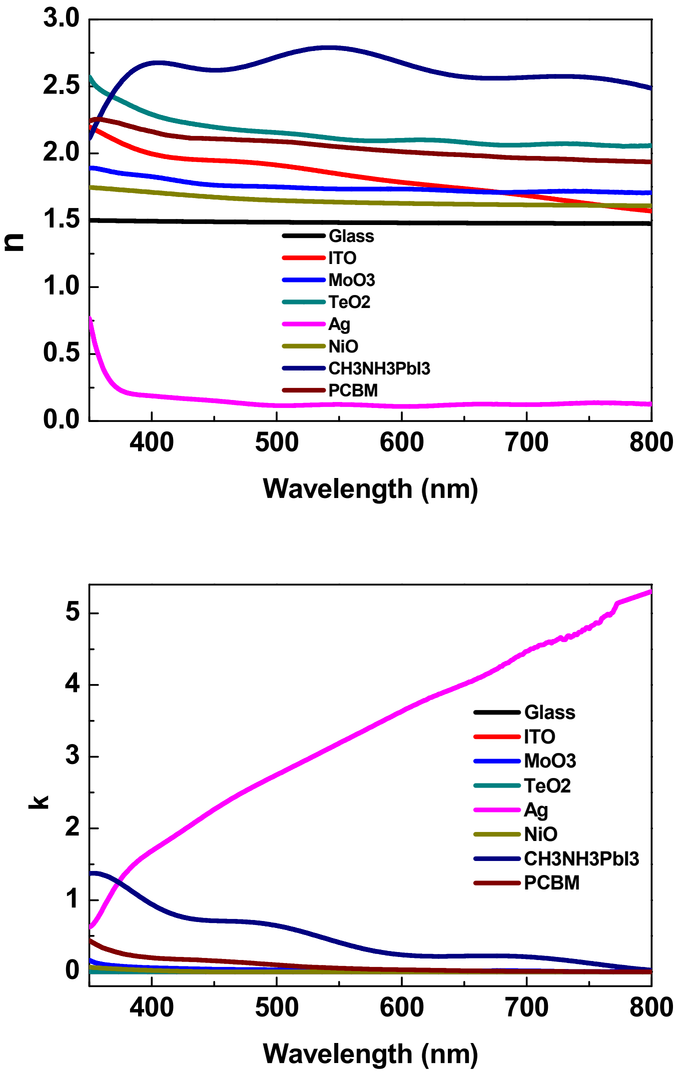


**Figure S1.** Refractive index of the materials used in the calculation.

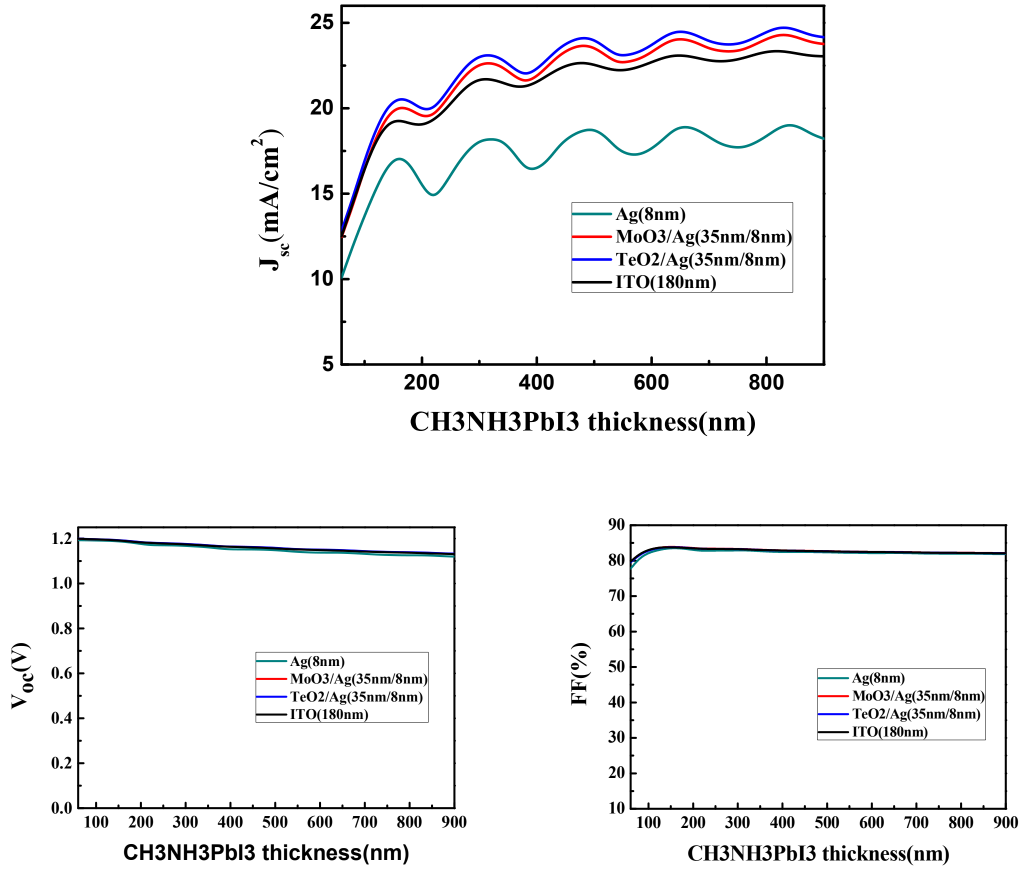


**Figure S2.** Variation of Jsc, Voc, FF for device D1 and device D2 with various $CH_3NH_3PbI_3$ thicknesses.

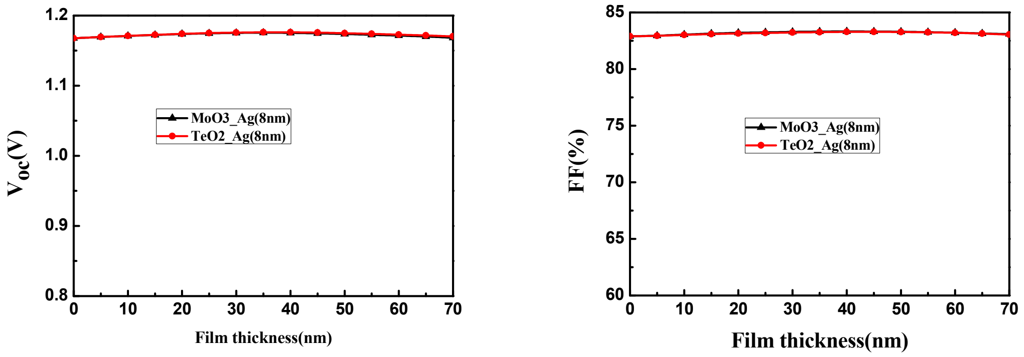


**Figure S3.** Variation of calculated $V_{OC}$ and FF with the different thicknesses of $MoO_3$ and $TeO_2$.

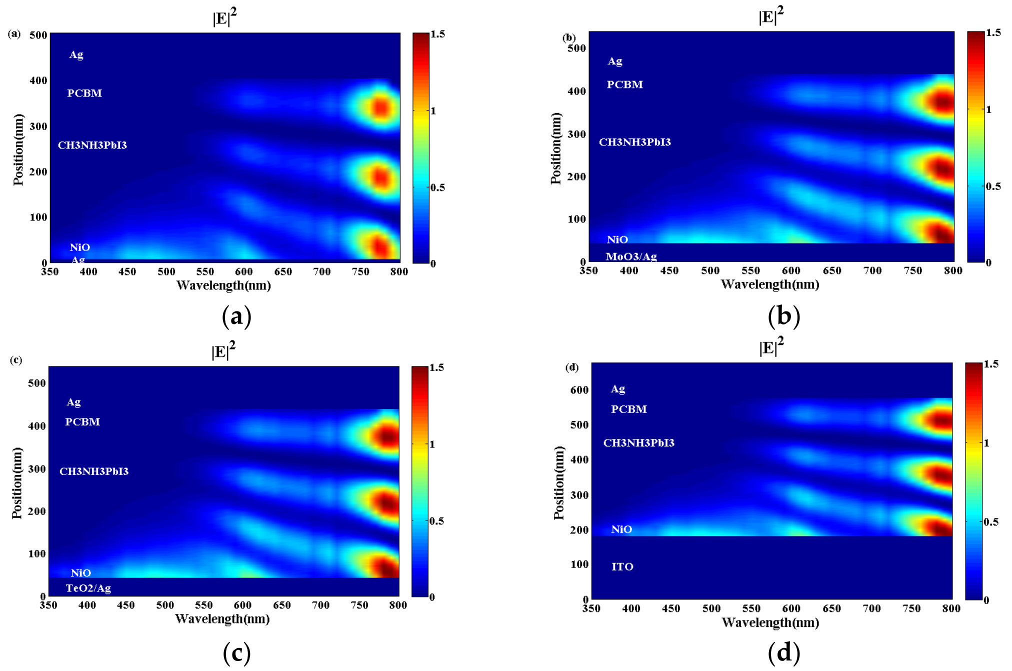


**Figure S4.** Optical electric field distribution for optical microcavity perovskite solar cells (PSCs) with (**a**) bare Ag (8 nm) electrode, (**b**) $MoO_3$/Ag (35 nm/8 nm) electrode, (**c**) $TeO_2$/Ag (35 nm/8 nm) electrode and (**d**) the conventional PSC with the indium-tin-oxide (ITO) electrode.

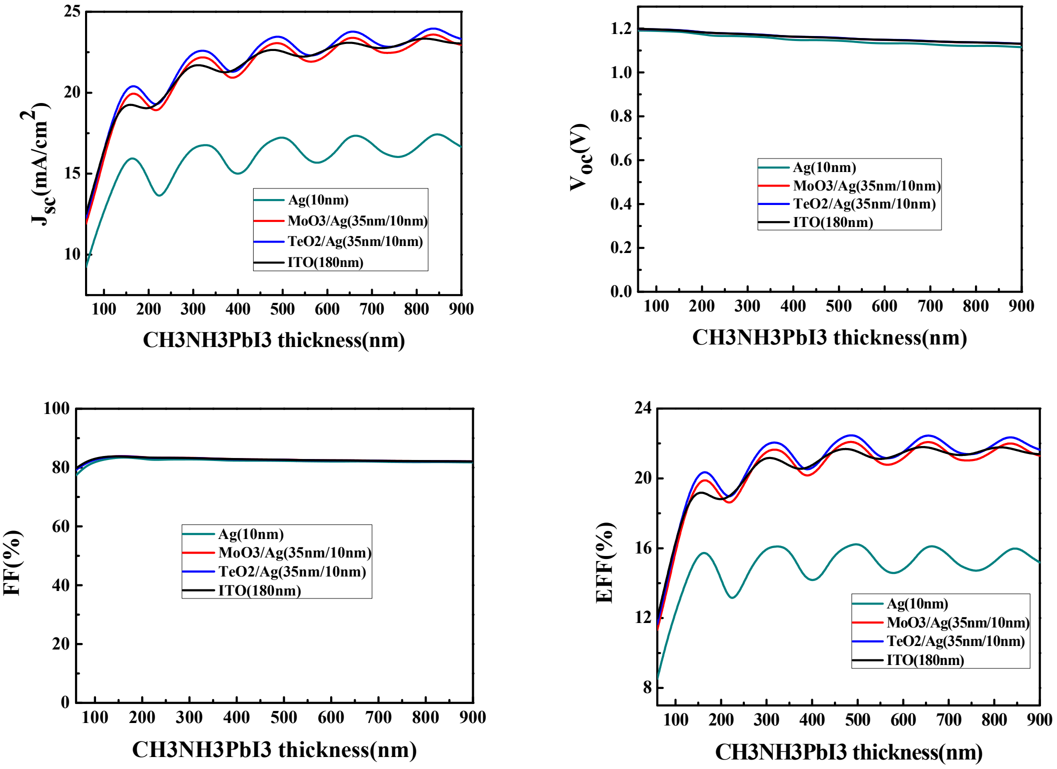


**Figure S5.** Variation of Jsc, Voc, FF and PCE for device D1 and device D2 with various $CH_3NH_3PbI_3$ thicknesses when the thickness of Ag is 10 nm.

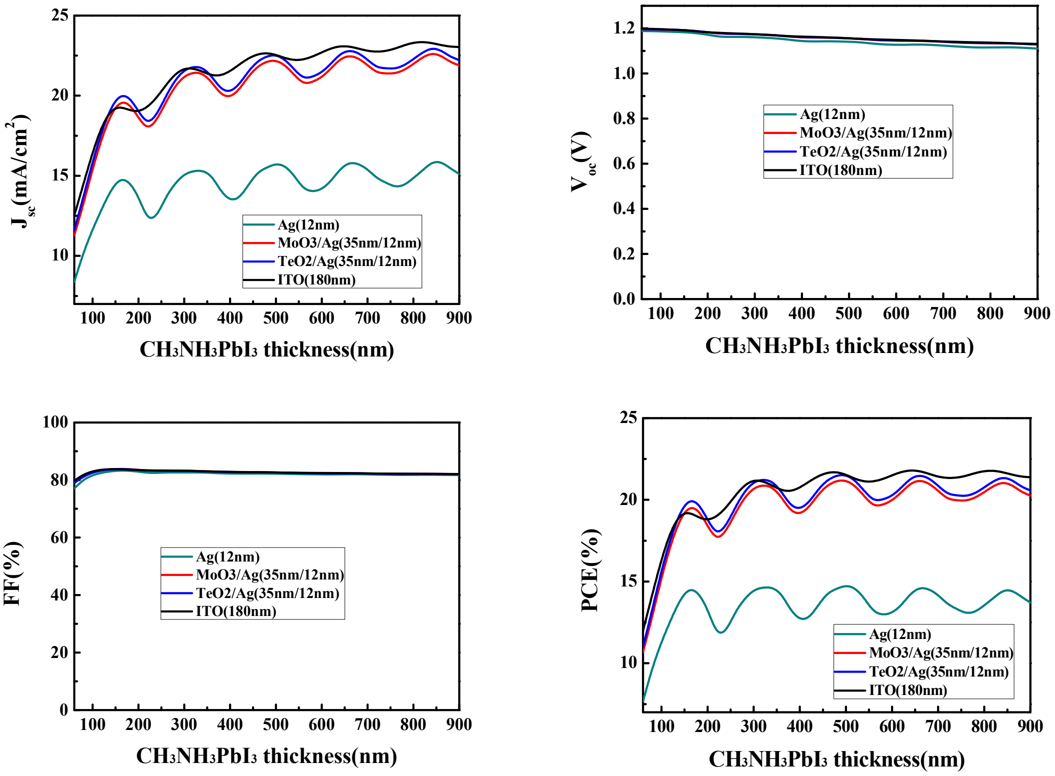


**Figure S6.** Variation of Jsc, Voc, FF and PCE for device D1 and device D2 with various $CH_3NH_3PbI_3$ thicknesses when the thickness of Ag is 12 nm.

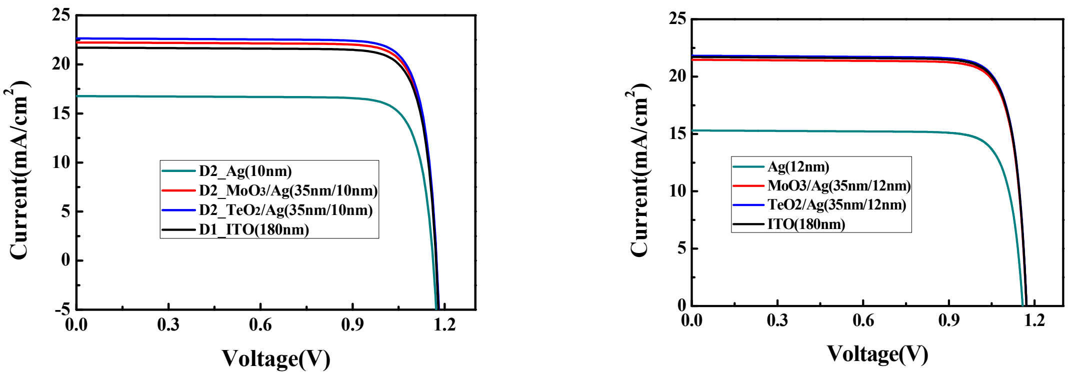


(**a**) (**b**)

**Figure S7.** I–V characteristic curves for the optical microcavity devices with the 10-nm Ag (**a**) and the 12-nm Ag (**b**).

© 2020 by the authors. Submitted for possible open access publication under the terms and conditions of the Creative Commons Attribution (CC BY) license (http://creativecommons.org/licenses/by/4.0/).
